# Identification of serum prognostic biomarkers of severe COVID-19 using a quantitative proteomic approach

**DOI:** 10.1038/s41598-021-98253-9

**Published:** 2021-10-19

**Authors:** Yayoi Kimura, Yusuke Nakai, Jihye Shin, Miyui Hara, Yuriko Takeda, Sousuke Kubo, Sundararaj Stanleyraj Jeremiah, Yoko Ino, Tomoko Akiyama, Kayano Moriyama, Kazuya Sakai, Ryo Saji, Mototsugu Nishii, Hideya Kitamura, Kota Murohashi, Kouji Yamamoto, Takeshi Kaneko, Ichiro Takeuchi, Eri Hagiwara, Takashi Ogura, Hideki Hasegawa, Tomohiko Tamura, Takeharu Yamanaka, Akihide Ryo

**Affiliations:** 1 Advanced Medical Research Center, Yokohama City University, Yokohama, 236-0004 Japan; 2 Department of Biostatistics, Yokohama City University School of Medicine, Yokohama, 236-0004 Japan; 3 Department of Microbiology, Yokohama City University School of Medicine, Yokohama, 236-0004 Japan; 4 Department of Pulmonology, Yokohama City University School of Medicine, Yokohama, 236-0004 Japan; 5 School of Medicine Medical Course Emergency Medicine, Yokohama City University, Yokohama, 236-0004 Japan; 6 Department of Respiratory Medicine, Kanagawa Cardiovascular and Respiratory Center, Yokohama, 236-0051 Japan; 7 Influenza Research Center, National Institute of Infectious Diseases, Musashimurayama, Tokyo 208-0011 Japan; 8 Department of Immunology, Yokohama City University School of Medicine, Yokohama, 236-0004 Japan

**Keywords:** Viral infection, Proteomic analysis, Biomarkers

## Abstract

The COVID-19 pandemic is an unprecedented threat to humanity that has provoked global health concerns. Since the etiopathogenesis of this illness is not fully characterized, the prognostic factors enabling treatment decisions have not been well documented. Accurately predicting the progression of the disease would aid in appropriate patient categorization and thus help determine the best treatment option. Here, we have introduced a proteomic approach utilizing data-independent acquisition mass spectrometry (DIA-MS) to identify the serum proteins that are closely associated with COVID-19 prognosis. Twenty-seven proteins were differentially expressed between severely ill COVID-19 patients with an adverse or favorable prognosis. Ingenuity Pathway Analysis revealed that 15 of the 27 proteins might be regulated by cytokine signaling relevant to interleukin (IL)-1β, IL-6, and tumor necrosis factor (TNF), and their differential expression was implicated in the systemic inflammatory response and in cardiovascular disorders. We further evaluated practical predictors of the clinical prognosis of severe COVID-19 patients. Subsequent ELISA assays revealed that CHI3L1 and IGFALS may serve as highly sensitive prognostic markers. Our findings can help formulate a diagnostic approach for accurately identifying COVID-19 patients with severe disease and for providing appropriate treatment based on their predicted prognosis.

## Introduction

Coronavirus disease 2019 (COVID-19) is a highly transmissible respiratory infection caused by the novel positive-sense, single-stranded RNA virus severe acute respiratory syndrome coronavirus 2 (SARS-CoV-2), which emerged in Wuhan, China in 2019. Despite containment efforts, rapid person-to-person transmission resulted in widespread dissemination and the disease has become a pandemic and is still spreading^1^. The molecular mechanisms of disease progression that cause respiratory distress in COVID-19 patients are still unknown, and no effective antiviral therapies for COVID-19 have been established to date^2^. In order to optimize allocations of limited health care resources to the neediest patients, it is crucial to accurately predict the progress and prognosis of patients with this disease. In addition, targeted management of high-risk patients will contribute to a further reduction in mortality^3,4^.

Most COVID-19 patients exhibit either mild symptoms without dyspnea or abnormal chest imaging, or moderate respiratory symptoms with pneumonia. They usually recover with or without supportive treatment. About 20% of patients develop respiratory distress and require immediate oxygen supplementation. A subset of these patients become critically ill, developing rapid respiratory failure and severe hypoxemia that necessitate immediate intensive care to prevent death. Considering the wide variety of clinical manifestations of COVID-19, identifying patients who are at risk of severe disease and adverse prognosis is crucial for selecting appropriate treatment strategies. For this purpose, pinpointing novel biological indicators that can serve as precise prognostic biomarkers is necessary to help clinicians make better clinical decisions and provide appropriate therapeutic strategies during earlier stages of the disease. To date, several clinical and biochemical parameters have been used to predict the severity of COVID-19, including the following: C-reactive protein (CRP), serum amyloid A (SAA), interleukin (IL)-6, lactate dehydrogenase (LDH), white blood cell count, d-dimer, cardiac troponin and platelet count^3,5^. In addition, multiple serological factors involved in the severity of COVID-19 have been identified by studies using a proteomic approach to analyze patient serum^6–10^. Most of these studies identified protein profiles involved in systemic and/or local inflammation, and that accompany organ damage or dysfunction. Although currently available serological biomarkers can predict severe disease, there are no reports of markers that predict the clinical prognosis and mortality of severe COVID-19 patients.

In this study, we utilized recently developed mass spectrometry technology with the data-independent acquisition (DIA-MS) approach to identify serum proteins closely associated with disease prognosis (discovery phase of the study). Using ELISA assays, we further delineated practical predictors of clinical prognosis in severe COVID-19 patients (verification phase of the study). Consequently, we identified two putative biomarkers that can indicate disease progression and adverse prognosis. These biomarkers shed light on a novel diagnostic approach that may serve to segregate COVID-19 patients based on their clinical prognosis and to select appropriate management measures.

## Results

### Use of quantitative proteomic analysis to identify serum proteins associated with favorable or adverse outcomes in severe COVID-19 patients

**Figure 1:**
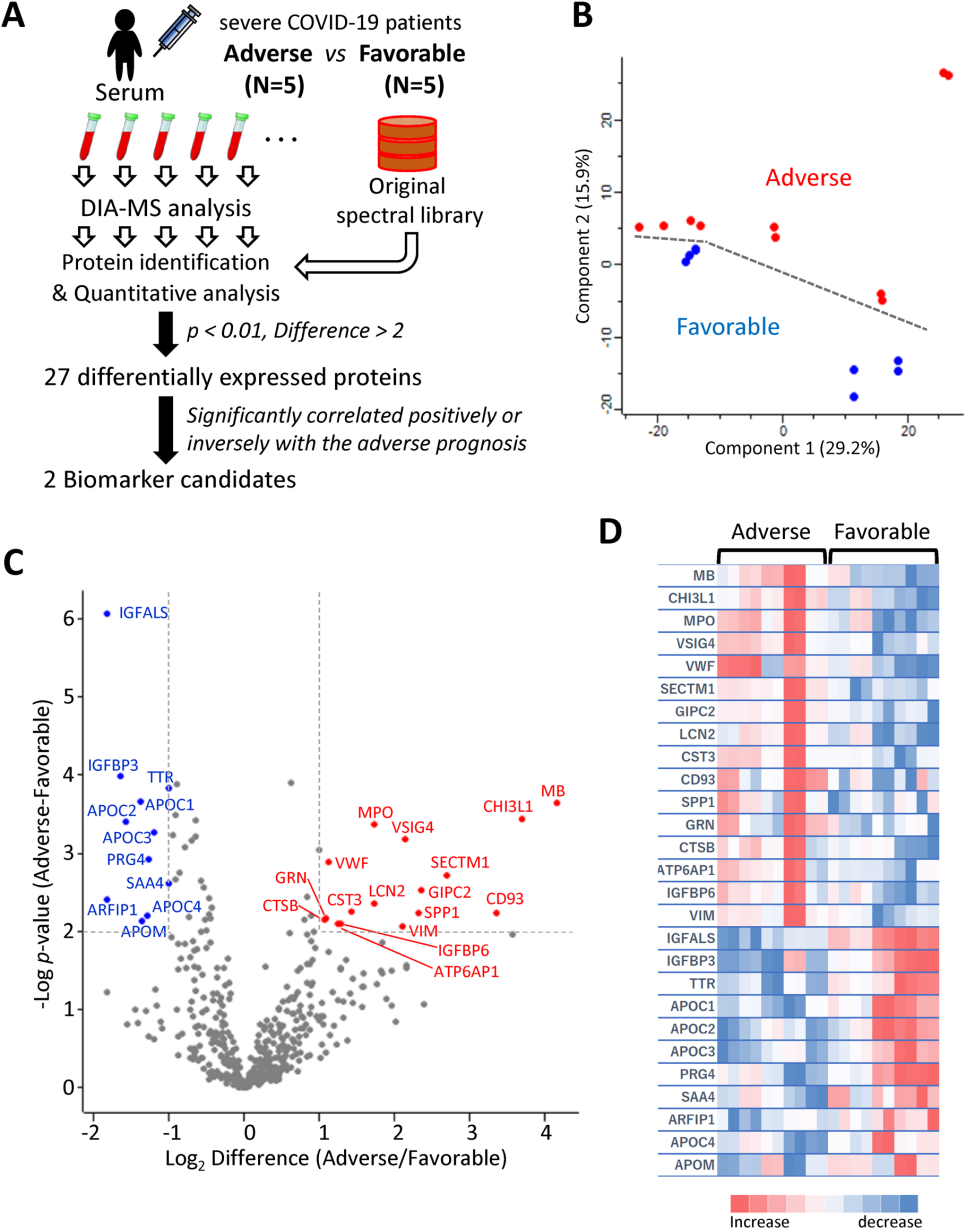
Serum protein profiling by DIA-MS analysis of severe COVID-19 patients with adverse or favorable prognosis. (**A**) Study design for identification of potential biomarker candidates. Serum protein profiling was performed by DIA-MS analysis discovery study comprising five patients each with adverse and favorable prognosis. (**B**) A PCA score plot for proteins in the two groups was generated using Perseus. Severe COVID-19 patients with adverse or favorable prognosis are represented by red and blue, respectively. (**C**) Volcano plot representing the difference in serum expression levels of 495 proteins between the two groups. Red or blue dots indicate proteins with increased or decreased serum expression levels, respectively, in patients with adverse prognosis compared to those with favorable prognosis (*p* < 0.01, difference [adverse/favorable] > 2) (listed in Table 1). (**D**) Heatmap visualization of the 27 differentially expressed proteins between severe COVID-19 patients with adverse or favorable prognosis. Darker colors indicate a greater increased or decreased effect (red for increased and blue for decreased). Gene names are shown on the left.

**Figure 2:**
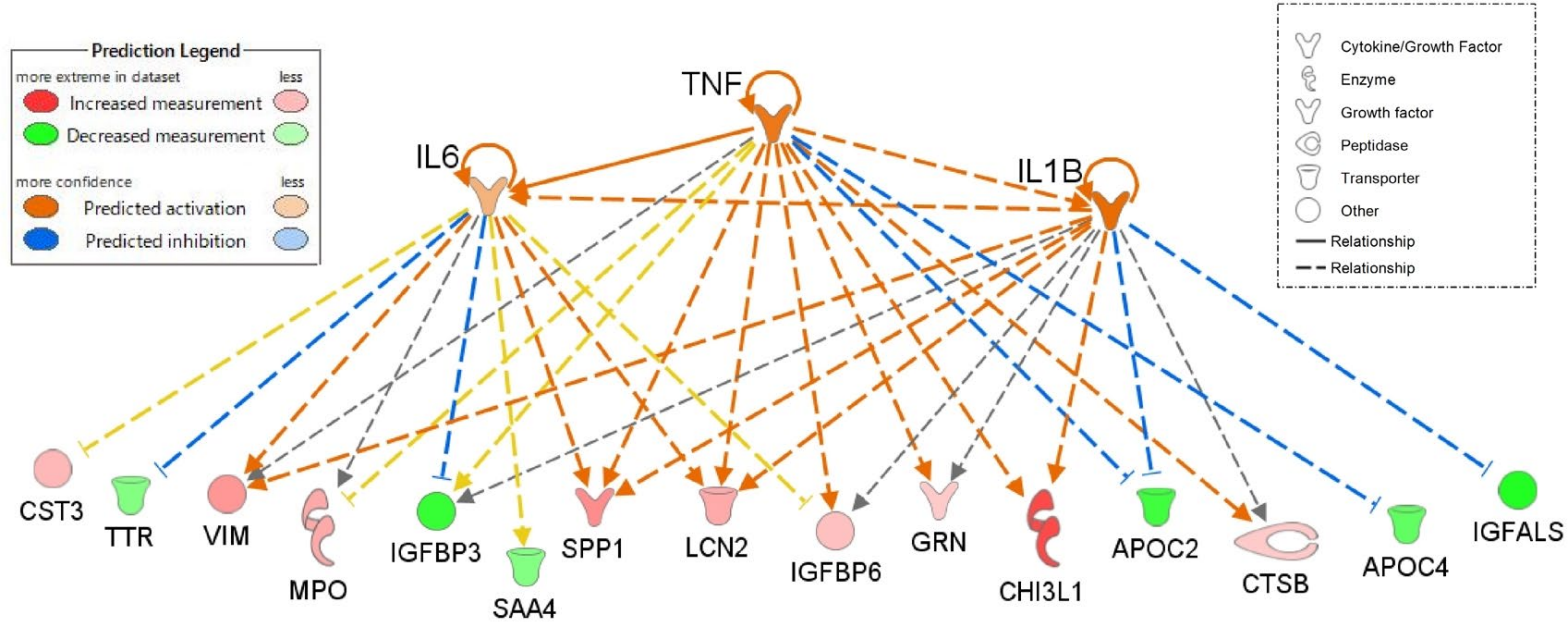
Network of proteins determined by biological upstream analysis within the framework of the IPA. The data was created using the IPA. Solid and dotted lines indicate direct and indirect interactions, respectively. Orange nodes represent molecules undetected in our study.

To identify serological biomarkers that predict a favorable or adverse prognosis in severe COVID-19 patients, we performed a comparative proteomic analysis with DIA-MS (Fig. 1A). In this discovery study, we obtained the MS data from serum samples collected within one day after the start of special inpatient intervention in 10 severe COVID-19 patients with different prognoses (five adverse and five favorable). By utilizing our customized spectral DIA library containing information on 1534 human serum proteins, we determined that 656 proteins were differentially expressed in sera. Among them, 495 proteins were selected for further statistical analysis (Supplementary Table S1). Subsequently principal component analysis (PCA) was used to visualize the distribution of the samples and revealed an obvious separation trend between the two groups (Fig. 1B). To identify proteins that differed markedly according to disease prognosis, volcano plot was used to analyze significant changes of proteins in severely ill patients with adverse prognosis. Consequently, 16 upregulated proteins and 11 downregulated proteins were identified as being significantly associated with an adverse COVID-19 prognosis (*p* < 0.01, fold change (difference) > 2) (Fig. 1C and Table 1). Indeed, a heatmap analysis exhibited hierarchical clustering of these proteins based on expression levels correlated with disease prognosis of severe COVID-19 patients (Fig. 1D). To investigate the biological processes affecting the severity of COVID-19, an upstream analysis was performed within the framework of Ingenuity Pathway Analysis (IPA). The results showed that several proteins in the sera of severely ill patients with adverse prognosis had increased or decreased levels and might be regulated by proinflammatory cytokines (Supplementary Table S2). Notably, out of 27 differentially expressed proteins, 15 were found to be regulated by IL-1β, IL-6, or tumor necrosis factor (TNF), which are seen at markedly higher levels in most severe COVID-19 patients^11,12^ (Fig. 2). In addition, a disease and functional enrichment analysis suggested that the several differentially expressed proteins could be associated with cardiovascular disorders (Supplementary Table S3). This result was consistent with the hypothesis that COVID-19 causes cardiovascular diseases, including myocardial injury and venous thromboembolism^13^. Simultaneously, there was evidence for inflammatory responses, such as neutrophil degranulation, as reported in the literature^7–10^. Furthermore, most of the 27 differentially expressed proteins formed an interconnected network, as revealed by the STRING database (Supplementary Fig. S1).

**Table 1:** Proteins differentially expressed in severe COVID-19 patients with adverse prognosis, compared to favorable prognosis.

Protein accessions	Genes	Protein descriptions	#Detected peptides	*p*-value (− log)	Difference [adverse/favorable] (log_2_)
**Increased protein**					
P02144	MB	Myoglobin	3	3.646	4.161
P36222	CHI3L1	Chitinase-3-like protein 1	5	3.433	3.690
P05164	MPO	Myeloperoxidase	7	3.363	1.725
Q9Y279	VSIG4	V-set and immunoglobulin domain-containing protein 4	2	3.185	2.147
P04275	VWF	von Willebrand factor	56	2.885	1.117
Q8WVN6	SECTM1	Secreted and transmembrane protein 1	1	2.724	2.690
Q8TF65	GIPC2	PDZ domain-containing protein GIPC2	1	2.526	2.356
P80188	LCN2	Neutrophil gelatinase-associated lipocalin	6	2.365	1.721
P01034	CST3	Cystatin-C	9	2.258	1.431
Q9NPY3	CD93	Complement component C1q receptor	3	2.232	3.348
P10451	SPP1	Osteopontin	5	2.232	2.321
P28799	GRN	Granulins	4	2.168	1.079
P07858	CTSB	Cathepsin B	3	2.146	1.063
Q15904	ATP6AP1	V-type proton ATPase subunit S1	1	2.106	1.252
P24592	IGFBP6	Insulin-like growth factor-binding protein 6	3	2.099	1.276
P08670	VIM	Vimentin	6	2.060	2.112
**Decreased protein**					
P35858	IGFALS	Insulin-like growth factor-binding protein complex acid labile subunit	24	6.068	− 1.829
P17936	IGFBP3	Insulin-like growth factor-binding protein 3	9	3.981	− 1.648
P02766	TTR	Transthyretin	17	3.832	− 1.002
P02654	APOC1	Apolipoprotein C-I	13	3.654	− 1.378
P02655	APOC2	Apolipoprotein C-II	3	3.399	− 1.567
P02656	APOC3	Apolipoprotein C-III	8	3.263	− 1.208
Q92954	PRG4	Proteoglycan 4	17	2.930	− 1.267
P35542	SAA4	Serum amyloid A-4 protein	5	2.621	− 1.003
P53367	ARFIP1	Arfaptin-1	1	2.407	− 1.825
P55056	APOC4	Apolipoprotein C-IV	5	2.206	− 1.291
O95445	APOM	Apolipoprotein M	5	2.139	− 1.363

### Identification of putative biomarkers for predicting the prognosis of severe COVID-19

**Figure 3:**
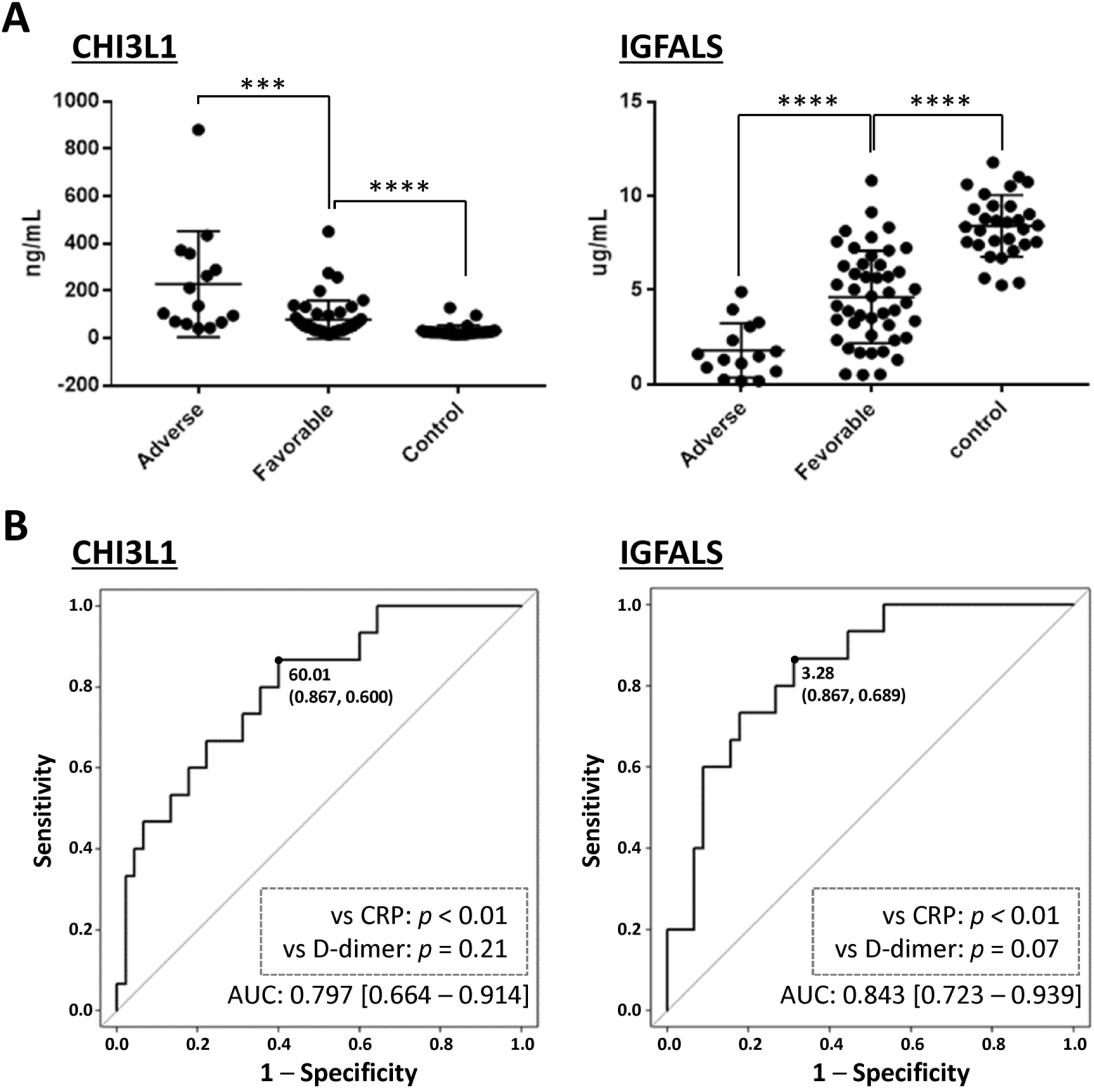
Serum levels and ROC curves of CHI3L1 and IGFALS as determined by ELISA assays. (**A**) In 15 COVID-19 patients with adverse prognosis, the median serum levels [interquartile range, IQR] of CHI3L1 and IGFALS were 136.2 [67.41–357.2] ng/mL and 1.479 [0.6873–3.059] µg/mL, respectively. In 46 COVID-19 patients with favorable prognosis, the median serum levels [IQR] of CHI3L1 and IGFALS were 49.87 [32.04–89.04] ng/mL and 4.498 [2.565–6.342] µg/mL, respectively. In 30 healthy controls, the median serum levels [interquartile range, IQR] of CHI3L1 and IGFALS were 24.3 [19.66–28.0] ng/mL and 8.396 [7.424–9.464] µg/mL, respectively. Differences in continuous values between the two groups were compared by the Mann–Whitney *U*-test: ***p < 0.001, ****p < 0.0001. These analyses were carried out with GraphPad Prism software. (**B**) ROC curves for predicting the clinical prognosis of severe COVID-19 patients. The optimal cut-off values (sensitivity and specificity) for making a clinical decision using CHI3L1 or IGFALS were defined as 60.01 ng/mL (0.867 and 0.600) and 3.28 µg/mL (0.867 and 0.689), respectively. The *p*-values were generated from bootstrap tests for the two ROC curves. AUC indicates the area under the ROC curve [95% CI].

To identify practical prognostic indicators in severe COVID-19 patients, we focused on two proteins, namely chitinase-3-like protein 1 (CHI3L1) and insulin-like growth factor-binding protein acid labile subunit (IGFALS), since statistical analyses showed that high and low levels of these proteins, respectively, were significantly correlated with adverse prognosis in severe COVID-19 patients (Table 1). We excluded myoglobin (MB) since it has previously been reported to be a prognostic marker^9^. To validate the clinical utility of these two prognostic marker candidates, we used ELISA assays to analyze the levels of these proteins in serum samples generally collected within three days after admission, except for four patients, in 61 severe COVID-19 patients (15 adverse and 46 favorable; Supplementary Tables S4 and S5). We also measured the levels of these proteins in the sera of healthy controls to compare them with the levels in severe COVID-19 patients with favorable prognosis. The clinical information and treatment of the recruited patients enrolled in the verification study is presented in Supplementary Tables S4 and S5. The analyzed parameters, excluding the administration of extracorporeal membrane oxygenation care and the incidence of death, did not differ between the adverse and favorable prognosis groups. On the other hand, ELISA assays of the serum levels of these proteins showed significant differences between severe COVID-19 patients with adverse versus favorable prognosis (*p* < 0.001) and between severe COVID-19 patients with favorable prognosis and healthy controls (*p* < 0.0001), suggesting that these protein levels correlate with adverse prognosis in severe COVID-19 patients (Fig. 3A). We further assessed the ability of CHI3L1 and IGFALS to predict adverse prognosis using receiver operating characteristic (ROC) curves. In the present study set, the areas under the ROC curves (AUCs) [95% confidence intervals (CI)] of models using CHI3L1 or IGFALS were 0.797 [0.644–0.914] and 0.843 [0.723–0.939], respectively (Fig. 3B). Internal validation of the final models for CHI3L1 and IGFALS was performed by bootstrap resampling, which showed that the two proteins maintained high predictive accuracy (optimism-adjusted AUC = 0.800 and 0.848, respectively). These values were more reliable than those of two established biomarkers, namely CRP (0.556 [0.375–0.736]) and d-dimer (0.688 [0.524–0.841]) (Supplementary Fig. S2). More precise statistical analysis using AUCs corroborated that CHI3L1 and IGFALS had higher reliability than CRP (Fig. 3B). Furthermore, the AUC [95% CI] of a model using both CHI3L1 and IGFALS was 0.862 [0.745–0.957]. Consequently, the serum expression levels of these two proteins can enhance clinical diagnostic accuracy by providing a precise indication of the outcome of severely ill COVID-19 patients.

## Discussion

COVID-19 manifests in numerous ways, ranging from a lack of symptoms leading to spontaneous recovery, to acute respiratory distress syndrome (ARDS) characterized by respiratory failure and diffuse alveolar damage^14^. While most patients with severe respiratory disorders recover successfully, a substantial number die of respiratory failure and/or systemic complications. Determining which individuals have the highest risk of adverse outcomes, including by identifying putative risk factors and/or biomarkers for severe illness, would be ideal for optimizing intensive medical management of COVID-19. For this purpose, we used the serum of COVID-19 patients to perform DIA-MS-based proteomic analysis, which has the potential to discover proteins previously not shown to be related to adverse prognosis. Consequently, we identified 27 candidate proteins whose serum levels were increased or decreased in patients with adverse prognosis. Subsequent statistical analysis using ROC curves found that two putative prognostic indicators, namely CHI3L1 and IGFALS, may be useful in severe COVID-19 patients.

Several studies have reported that most severe COVID-19 patients exhibit marked increases in serum levels of proinflammatory cytokines^11,12,15^. Therefore, the current understanding of the disease suggests that cytokine storm, along with the immunological dysregulation triggered by the viral replication phase, contributes to the progression of severe ARDS and multiple organ failure in COVID-19^11,16^. However, IL-6 levels in COVID-19 patients are lower than the median values typically reported in ARDS^17,18^, and other unidentified determinants may define COVID-19 severity. In this study, we investigated the molecular relevance of CHI3L1 and IGFALS by an upstream analysis using the IPA framework. This showed that the expression levels of these proteins were regulated by proinflammatory cytokines such as IL-1β, IL6, or TNF, indicating that our new biomarkers could be markers for the proinflammatory cytokine network and cascade.

CHI3L1, also termed YKL-40, is a protein that binds with chitin but lacks chitinase activity. We found that serum CHI3L1 levels were elevated in COVID-19 patients with severe disease and adverse prognosis. Parallel to this finding, previous studies show that high serum levels of CHI3L1 are associated with an increased risk of death from various causes, including cardiovascular disease^19,20^. Additionally, elevated serum levels of CHI3L1 were found in patients with idiopathic pulmonary fibrosis (IPF)^21^. Immunohistochemistry also showed that the expression levels of CHI3L1 were enhanced in bronchiolar epithelial cells and alveolar macrophages adjacent to fibrotic lesions in patients with IPF, suggesting the possible involvement of CHI3L1 in the fibrotic process of IPF^21^. These findings together suggest that CHI3L1 plays an important role in tissue remodeling of the respiratory system in COVID-19^22,23^. Therefore, higher levels of CHI3L1 might be associated with the pathogenesis of COVID-19, especially in terms of pulmonary tissue damage and repair.

This study also demonstrated the reduction of IGFALS levels in severe COVID-19 patients with adverse prognosis. In healthy individuals, IGFALS forms a ternary complex with IGFBP3 and insulin-like growth factor 1 (IGF-1). The binding of IGFALS/IGFBP3 to IGF-1 has been shown to prevent the interaction of IGF-1 with its receptor, IGF-1R, and to reduce the stability of IGF-1 and thereby suppress its biological function^24^. It was also observed that plasma levels of IGF-1 were significantly reduced in mice with complete deficiency of IGFALS, suggesting an accelerated reduction of the half-life of IGF-1 despite no changes in its liver or renal expression^25,26^. Consequently, deficiency of IGFALS disrupts IGF-1 circulation without affecting glucose or insulin homeostasis^25^. The role of IGF-1 signaling in fibrotic processes varies depending on spatial and stoichiometric conditions^27^. Irrespective of COVID-19, IGF-1 levels diminish gradually in later fibroproliferative stages, and show a negative correlation with mortality in patients with ARDS^28,29^. Moreover, a recent study indicated that low serum IGF-1 levels were associated with a higher risk of mortality in COVID-19 patients^30^. Together these findings may suggest that serum IGF-1/IGFALS levels are directly or indirectly involved in respiratory dysfunction. However, the regulatory mechanisms of IGFALS and IGF-1 in COVID-19 remain elusive, and further studies are needed to determine the functional roles of both proteins in the pathogenesis of the disease.

The findings of this study may enhance the ability to identify which COVID-19 patients with severe pneumonia are at high risk of mortality, based on the serum levels of two proteins closely involved in the pathogenesis in COVID-19. The ability of CHI3L1 and IGFALS to discriminate favorable and adverse prognosis in COVID-19 patients was superior to that of the existing biomarkers.

This study has some limitations, especially in the initial discovery study, where we used only a small sample size to detect the differentially expressed proteins between the adverse and favorable prognosis groups. Also, the use of a COVID-19 patient specific spectral DIA library would be a more ideal representation than the customized spectral library that we had created to discover new biomarkers. The use of the former would probably identify more proteins altered during COVID-19 infection. However, despite these shortcomings of the discovery study, the verification study revealed that the newly discovered biomarkers CHI3L1 and IGFALS correlated better with the prognostic outcomes than the currently existing biomarkers. Further prospective studies with a larger sample size are needed to validate the quality of these biomarkers. A multidisciplinary approach and a multivariable statistical analysis of these biomarkers will be useful for determining their ability to predict the clinical prognosis of severe COVID-19.

## Methods

### Human samples

Serum samples were obtained from COVID-19 patients who were hospitalized at Yokohama City University Hospital, Yokohama City University Medical Center, and National Hospital Organization Yokohama Medical Center from February 2020 to January 2021, or from otherwise healthy volunteers (employees of Yokohama City University) from January 2014 to December 2015. This research protocol was approved by the Clinical Ethics Committee of Yokohama City University Hospital (B2002000048 and B160800009). This study was also performed with the approval of the Clinical Ethics Committee of each participating medical facility. Informed consent was obtained from all patients or their guardians before serum sample collection. This study was conducted in accordance with the Declaration of Helsinki. All the data was anonymized before the analyses.

All patients in the study were diagnosed with COVID-19 according to the Manual for the Detection of Pathogen 2019-nCoV of the National Institute of Infectious Diseases in Japan. Severe COVID-19 patients were defined according to the National Institutes of Health guidelines. In addition, patients with severe disease who died or required extracorporeal membrane oxygenation were classified as having adverse prognosis, while the remainder were defined as having favorable prognosis. All serum samples were stored at − 80 °C until use and then denatured by adding an equal volume of 8 M urea solution for MS analysis.

### LC mass spectrometry

Desalted peptides were resuspended in 0.1% formic acid and 2% ACN containing iRT peptides (Biognosys) and then analyzed using a Q-Exactive mass spectrometer coupled with an UltiMate 3000 HPLC system (Thermo Fisher Scientific). The mass spectrometer was operated using Xcalibur software. Peptides were loaded on a trap column (100 μm × 20 mm, C18, 5 μm, 100 Å, Thermo Fisher Scientific) and subsequently separated on a Nano HPLC capillary column (75 μm × 180 mm, C18, 3 μm, Nikkyo Technos) at a flow rate of 300 nL/min. Solvent A was 0.1% formic acid in 2% acetonitrile (ACN), while solvent B was 0.1% formic acid in 95% ACN. Peptides were eluted using a gradient from 2% B for 0–5 min, then 2–33% B for 5–120 min followed by 90% B for 10 min, and finally equilibrated for 20 min at 2% B. Data were acquired using either data-dependent acquisition (DDA) or data-independent acquisition (DIA).

### Human sera spectral library generation

For the comprehensive serum proteome analysis, we attempted to construct an original DIA-MS system for human serum. To construct a serum spectral library, four different human pooled sera purchased from Kohjin Bio (cat# 12181201), Biowest (cat# S4200), PAN-Biotech (cat# P30-2701), and Sigma-Aldrich (cat# S7023) were used because of the limited amount of serum from COVID-19 patients. These sera were pooled and then fractionated in three ways, as described below, after removal of 14 human proteins (albumin, IgG, antitrypsin, IgA, transferrin, haptoglobin, fibrinogen, alpha2-macroglobulin, alpha1-acid glycoprotein, IgM, apolipoprotein AI, apolipoprotein AII, complement C3, and transthyretin) using a Human 14 Multiple Affinity Removal System (MARS) column (Agilent Technologies) or after compression of the dynamic range of protein abundance using ProteoMiner beads (Bio-Rad). First, the immunodepleted or compressed serum was fractionated using an HPLC system with a C4 reversed-phase column (Vydac), and 20 fractions were independently subjected to in-solution digestion with trypsin (Promega)^31^. Second, the immunodepleted or compressed serum was separated using a 5–20% polyacrylamide gel and then the gel was fractionated into six sections, followed by in-gel digestion with trypsin^32^. Third, the immunodepleted serum was digested with trypsin, and the resultant peptides (240 μg) were separated into 24 fractions using a 3100 OFFGEL Fractionator^33^. After desalting using a Stage Tip^34^, the obtained peptides were analyzed in DDA mode. The Q-Exactive was set to positive mode in a top-20 configuration. DDA mode analytical conditions consisted of a full MS1 scan with a resolution of 70,000 and a scan range from 350 to 1500 *m/z*, with the automatic gain control (AGC) target value being set to 3e^6^ (Full MS) and 1e^5^ (MS/MS). The normalized collision energy was set to 27. Spectral library generation from a data set containing 76 DDA-MS measurements was performed using Spectronaut Pulsar X (Ver.12.0.2, Biognosys) by searching against the iRT fasta database (Biognosys) and human protein sequences from the UniProtKB/Swiss-Prot database (version January 28, 2019), allowing for variable N-terminal acetylation, N-terminal carbamylation, methionine oxidation, and cysteine carbamidomethylation. MS1 and MS2 tolerances were set to dynamic, and two missed cleavages were allowed. Search results were filtered to satisfy a false discovery rate (FDR) of 1% on peptide levels and 5% on protein levels using Spectronaut Pulsar X for identification.

### Sample preparation for DIA-MS analysis

After adding 20 ng/μl *E. coli *β-galactosidase (β-gal) as the internal standard, 14 high abundance serum proteins (albumin, IgA, IgD, IgE, IgG, IgG [light chains], IgM, alpha-1-acid glycoprotein, alpha-1-antitrypsin, alpha-2-macroglobulin, apolipoprotein A1, fibrinogen, haptoglobin, and transferrin) were removed using High Select Top14 Abundant Protein Depletion Mini Spin Columns (Thermo Fisher Scientific). After centrifugal ultrafiltration using Amicon Ultra centrifugal filters, immunodepleted serum samples were dissolved in 4 M urea solution. To determine the reproducibility of results obtained using immunodepletion column, the proteins separated by SDS-PAGE were transferred to polyvinylidene fluoride membranes and then incubated with anti-β-gal antibody (diluted 1:1000) at room temperature (data not shown). Subsequently, proteins in 2 µl of immunodepleted serum were reduced with DTT (final concentration of 10 mM) and alkylated with 2-iodoacetamide (final concentration of 25 mM). The protein solutions were diluted from 8 to 2 M urea in 50 mM NH_4_HCO_3_ and then incubated with trypsin (final concentration, 15 ng/μl) at 37 °C for 16 h. To prepare the resultant peptides for MS analysis, they were desalted using a Stage Tip^34^, and the subsequently eluted peptides were completely lyophilized and kept at − 80 °C until use.

### DIA-MS analysis and data analysis

To determine protein abundance, serum peptide samples were analyzed twice each in DIA mode. DIA mode analytical conditions consisted of a full MS1 scan with a resolution of 70,000 full width at half maximum (FWHM) with a scan range from 380 to 1240 *m/z*, with the AGC target value being set to 3e^6^, followed by 40 DIA windows acquired at a resolution of 35,000 FWHM, with the AGC target value being set to 3e^6^. The isolation width and normalized collision energy were set to 5 *m/z* and 28, respectively. DIA-MS data were analyzed using Spectronaut Pulsar X against the spectral library to identify and quantify peptides and proteins. The retention time among different samples was calibrated using the iRT peptides. The Biognosys default settings were applied for identification; duplicate assays were excluded and FDRs were estimated using a *q*-value of 0.01 for both precursors and proteins. Interference correction was activated and a minimum of three fragment ions and two precursor ions were kept for the quantification. The area of the extracted ion chromatogram at the MS/MS level was used for quantification. Peptide quantity was measured by the mean of the 1–10 best precursors, and protein quantity was calculated accordingly by summing the 1–10 best peptides. The global normalization strategy and q-value sparse selection were used for cross run normalization. All other settings were set to their defaults. To perform downstream statistical quantitative analysis, we used Perseus (Max-Planck-Institute of Biochemistry), which is a software program for functional analysis of large-scale quantitative data^35^. Distinct samples were categorized into two groups, the intensity values were log_2_-transformed, and only proteins present in at least 70% of samples in each group were used for further analysis. The missing values were replaced by random numbers drawn from a normal distribution with a value of 0.3 for the width parameter and 1.8 for the down-shift parameter. A PCA score plot and volcano plot were created with Perseus. Protein interaction analysis was carried out with the online tool STRING (https://string-db.org, default settings)^36^. IPA (Content version: 60467501, Release Date: 2020-11-19, QIAGEN) was used for the biological analysis. ELISA assays were performed to measure the serum levels of CHI3L1 (cat# CY8088V2, MBL) and IGFALS. The ELISA assay for IGFALS was constructed using two anti–human IGFALS antibodies (cat# 537302 and cat# 537404, BioLegend) and a recombinant human IGFALS/ALS protein (cat# 9917-IA-050, R&D). ROC curve analysis was performed to assess the predictive performance of CHI3LI, IGFALS, d-dimer and CRP. The optimal cut-off value was determined by Youden’s index. Internal validation was performed by bootstrapping and was done with 150 simulations to obtain a bootstrapped AUC.

Statistical analysis was performed using GraphPad Prism software (version 7.0.2) or statistical software R (version 4.0.2).

## Supplementary Information

Supplementary Figures.

Supplementary Table 1.

## Data Availability

All mass spectrometry proteomics data have been deposited to the ProteomeXchange Consortium (http://www.proteomexchange.org,) via the jPOST (https://jpostdb.org) partner repository with the dataset identifier PXD027635 (Spectral library data) or PXD021702 (DIA-MS analysis data). All data are fully available without restriction.
