# Supplementary material for: Identification of serum prognostic biomarkers of severe COVID-19 using a quantitative proteomic approach
Source: Sci Rep. 2021 Oct 19;11:20638. doi: 10.1038/s41598-021-98253-9 (PMC8526747; doi:10.1038/s41598-021-98253-9)
Supplement: Supplementary file 1 — Supplementary Figures. [file 41598_2021_98253_MOESM1_ESM.pptx]

## Slide 1
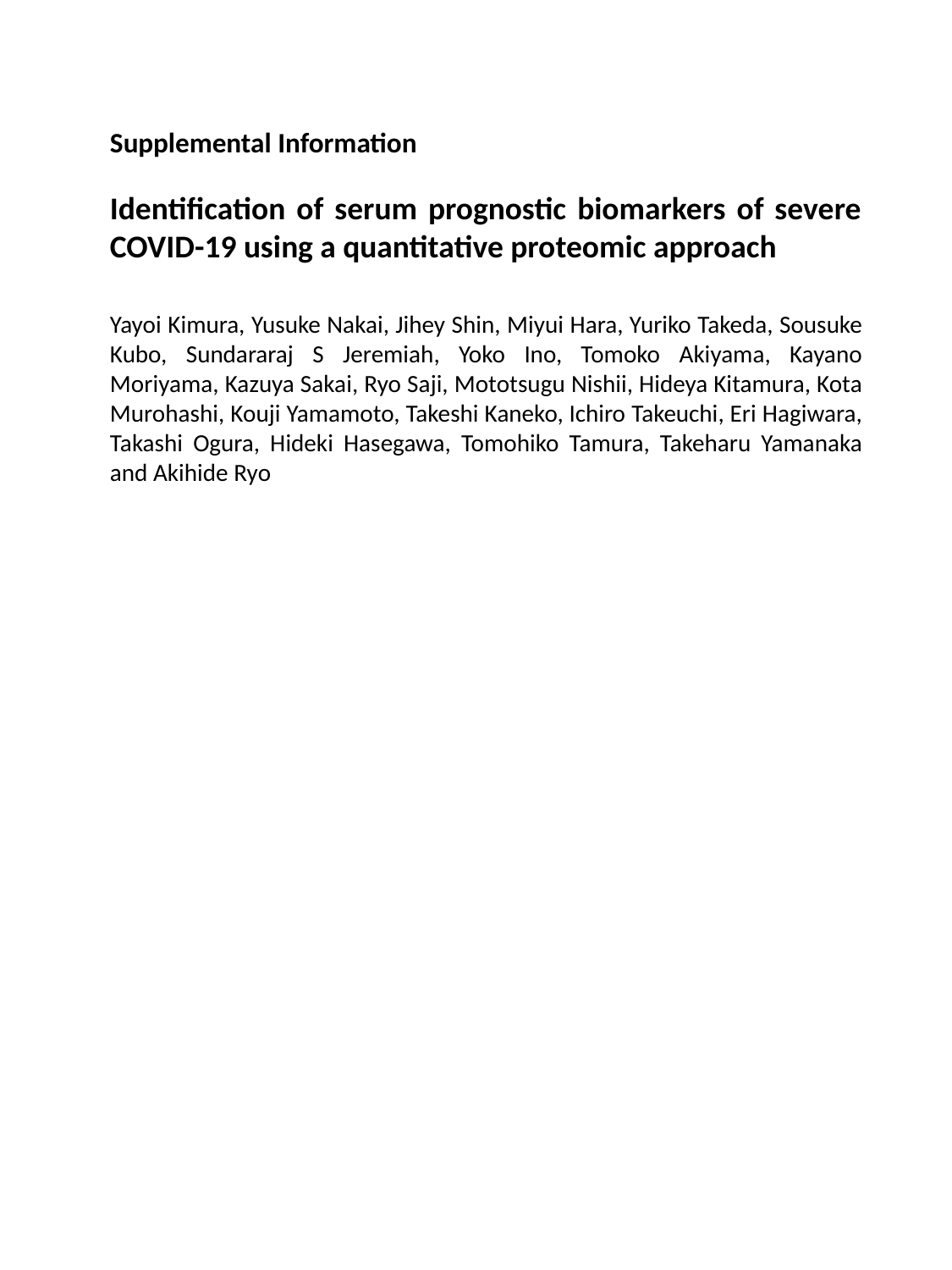

Supplemental Information
Identification of serum prognostic biomarkers of severe COVID-19 using a quantitative proteomic approach
Yayoi Kimura, Yusuke Nakai, Jihey Shin, Miyui Hara, Yuriko Takeda, Sousuke Kubo, Sundararaj S Jeremiah, Yoko Ino, Tomoko Akiyama, Kayano Moriyama, Kazuya Sakai, Ryo Saji, Mototsugu Nishii, Hideya Kitamura, Kota Murohashi, Kouji Yamamoto, Takeshi Kaneko, Ichiro Takeuchi, Eri Hagiwara, Takashi Ogura, Hideki Hasegawa, Tomohiko Tamura, Takeharu Yamanaka and Akihide Ryo

## Slide 2
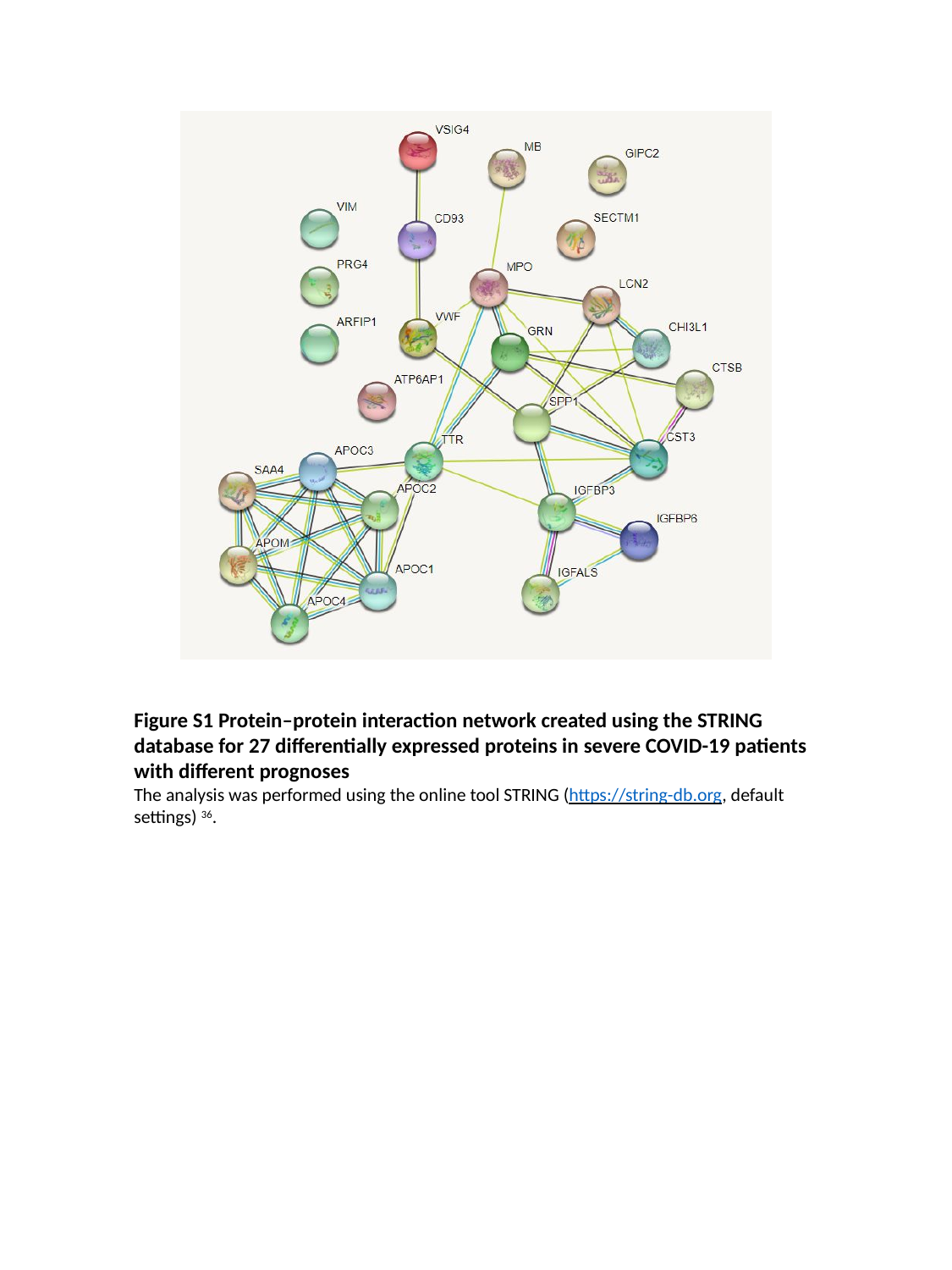

Figure S1 Protein–protein interaction network created using the STRING database for 27 differentially expressed proteins in severe COVID-19 patients with different prognoses
The analysis was performed using the online tool STRING (https://string-db.org, default settings) 36.

## Slide 3
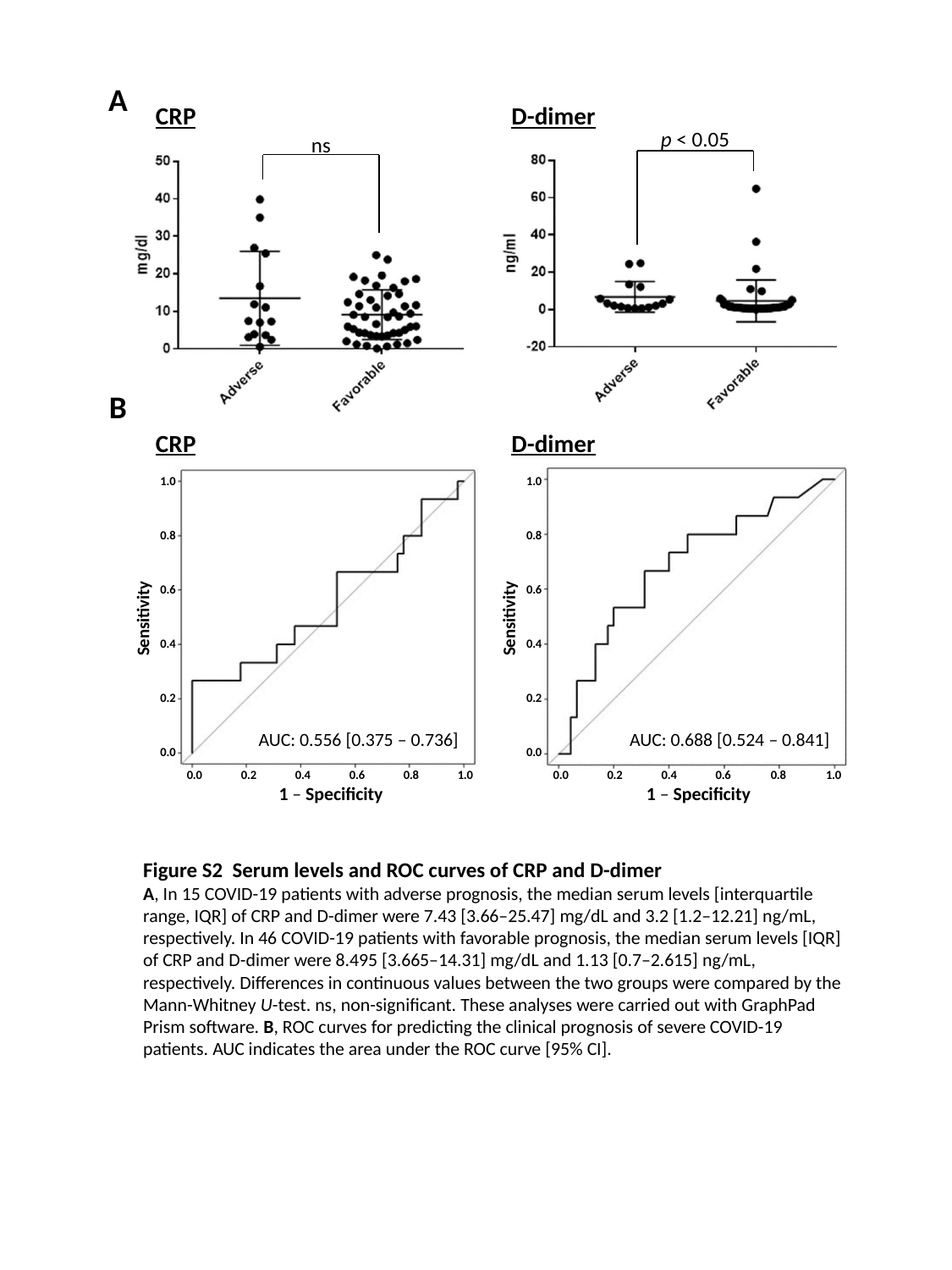

A
D-dimer
CRP
p < 0.05
ns
B
D-dimer
CRP
1.0
0.8
0.6
Sensitivity
0.4
0.2
0.0
1.0
0.8
0.6
Sensitivity
0.4
0.2
0.0
AUC: 0.556 [0.375 – 0.736]
AUC: 0.688 [0.524 – 0.841]
0.0
0.2
0.4
0.6
0.8
1.0
1 – Specificity
0.0
0.2
0.4
0.6
0.8
1.0
1 – Specificity
Figure S2 Serum levels and ROC curves of CRP and D-dimer
A, In 15 COVID-19 patients with adverse prognosis, the median serum levels [interquartile range, IQR] of CRP and D-dimer were 7.43 [3.66–25.47] mg/dL and 3.2 [1.2–12.21] ng/mL, respectively. In 46 COVID-19 patients with favorable prognosis, the median serum levels [IQR] of CRP and D-dimer were 8.495 [3.665–14.31] mg/dL and 1.13 [0.7–2.615] ng/mL, respectively. Differences in continuous values between the two groups were compared by the Mann-Whitney U-test. ns, non-significant. These analyses were carried out with GraphPad Prism software. B, ROC curves for predicting the clinical prognosis of severe COVID-19 patients. AUC indicates the area under the ROC curve [95% CI].
